# Fractionated stereotactic radiotherapy in people with drug-resistant focal epilepsy: first-in-human experience with a healthy tissue-preserving dose-fractionation concept

**DOI:** 10.3389/fneur.2025.1600381

**Published:** 2025-06-26

**Authors:** Cas Stefaan Dejonckheere, Attila Rácz, Gustavo Renato Sarria, Julian Philipp Layer, Younèss Nour, Lara Caglayan, Molina Grimmer, Victoria Volkenborn, Fabian Kugel, Thomas Müdder, Tobias Baumgartner, Valeri Borger, Alexander Radbruch, Hartmut Vatter, Frank Anton Giordano, Eleni Gkika, Rainer Surges, Davide Scafa

**Affiliations:** ^1^Department of Radiation Oncology, University Hospital Bonn, Bonn, Germany; ^2^Department of Epileptology, University Hospital Bonn, Bonn, Germany; ^3^Institute of Experimental Oncology, University Hospital Bonn, Bonn, Germany; ^4^Department of Neurosurgery, University Hospital Bonn, Bonn, Germany; ^5^Department of Neuroradiology, University Hospital Bonn, Bonn, Germany; ^6^Department of Radiation Oncology, University Medical Center Mannheim, Mannheim, Germany; ^7^DKFZ-Hector Cancer Institute, University Medical Center Mannheim, Mannheim, Germany; ^8^Medical Faculty Mannheim, Mannheim Institute of Intelligent Systems in Medicine (MIISM), Heidelberg University, Mannheim, Germany

**Keywords:** epileptic seizures, fractionated stereotactic radiotherapy, stereotactic radiosurgery, non-invasive, epilepsy

## Abstract

**Objective:**

Stereotactic radiosurgery (SRS) emerges as a non-surgical treatment option for drug-resistant non-neoplastic focal epilepsy. Previous studies have reported that in about 20% of patients treated with radiotherapy, however, subsequent salvage surgery is required, among other because of symptomatic radiation necrosis (RN). We propose a novel and radiobiologically substantiated dose-fractionation regimen which minimizes the RN risk while aiming to preserve efficacy and report our first-in-human experience.

**Methods:**

From February 2021 to April 2024, three patients (aged 42, 45, and 47 years) with different underlying etiologies were treated, including a post-hemorrhagic lesion, Rasmussen encephalitis, and focal cortical dysplasia. We applied linac-based frameless fractionated stereotactic radiotherapy (fSRT) to a total dose of 50 Gy in 10 fractions over 2 weeks. Each epileptogenic zone was defined by a multidisciplinary team, including a radiation oncologist, epileptologist, neurosurgeon, and neuroradiologist.

**Results:**

The irradiated volumes were 10.3, 11.3, and 16.5 cm^3^. After a follow-up of 12, 29, and 36 months, all three patients experienced an improvement in both seizure frequency and severity (two already during or shortly after fSRT). One patient achieved complete seizure freedom. All patients reported improvements in quality of life and regained independence or displayed functional recovery. Tolerability was excellent, with radiation-induced side effects being mild (grade 1 only) and transient. RN was not observed. One patient died 29 months after radiotherapy most likely from a ruptured aneurysm of a vertebral artery, unrelated to the treatment.

**Significance:**

Frameless fSRT of 50 Gy in 10 fractions was feasible and might be safe and effective in selected patients with drug-resistant non-neoplastic focal epilepsy and large suspected epileptogenic zones. A prospective single-arm evaluation with structured long-term follow-up including assessment of patient-reported outcome measures is currently being conducted.

## Highlights

- Radiotherapy is an effective treatment option for drug-resistant focal epilepsy.
- Limited by risk of radiation necrosis, especially if large epileptogenic zone.
- First-in-human experience with frameless stereotactic radiotherapy of 50 Gy in 10 fractions, reducing radiation necrosis risk.
- Novel concept is feasible, safe, and effective, no radiation necrosis observed.
- Prospective validation in larger sample size required.

## 1 Introduction

Epilepsy, one of the most common neurological conditions worldwide, has an estimated prevalence of 6 per 1,000 persons, with higher numbers reported in low- and middle-income countries (1). Recurrent seizures are known to negatively impact quality of life and impose a significant burden on patients, caregivers, and healthcare systems (2, 3). Furthermore, patients are at risk of injury as well as premature (unexpected) death, often stemming from associated comorbidities (4, 5). First-line treatment includes daily intake of antiseizure medication (ASM), successfully suppressing seizures in up to two-thirds of patients (6). In those with drug resistance or unmanageable ASM side effects, surgery poses an effective and potentially curative, yet underused, treatment option (6, 7). A smaller, but significant proportion of patients, however, are considered to have drug-resistant and inoperable epilepsy, either due to the location of the primary seizure-causing lesion (i.e., the epileptogenic zone comprises or is close to an eloquent area), contraindications for surgery (medical or functional inoperability), or patient wish not to undergo surgery.

In this selected collective, radiotherapy has been proposed as a promising non-invasive treatment alternative. A 2018 systematic review by Eekers et al. (8) identified 16 studies with a total of 170 adult patients who underwent stereotactic radiosurgery (SRS; majority of trials, dose ranging from 13–25 Gy) or fractionated stereotactic radiotherapy (fSRT; heterogeneous concepts) for drug-resistant non-neoplastic focal epilepsy. Focusing on frequency of seizures as the primary outcome, an average (range) of 58% (25–95%) of patients reported either no or rare seizures following SRS or fSRT. In one-fifth of patients, however, subsequent salvage surgery was required (8). Reasons included the development of cysts, edema, intracranial hypertension, remaining seizures, or radiation necrosis (RN). The latter is a known late complication of radiotherapy, most likely as a result from vascular changes following radiation-induced endothelial injury (9, 10). The risk of RN depends primarily on the volume of irradiated brain tissue [e.g., expressed by the volume receiving 12 Gy in single-fraction treatments (V_12Gy_)], with a higher frequency after SRS than fSRT (8–11).

As epilepsy is a chronic condition and life expectancy is usually significantly superior to oncological radiotherapy indications (e.g., brain metastases), an accurate assessment of potential risks and benefits is required when considering this treatment option. As high-level evidence on the effects of radiotherapy for drug-resistant non-neoplastic focal epilepsy is currently being gathered, we propose a novel and radiobiologically substantiated dose-fractionation regimen which minimizes the RN risk while aiming to preserve radiotherapy efficacy. Here, we report our preliminary experience in a case series of three patients.

## 2 Materials and methods

### 2.1 Patient selection

From the institutional database, we retrospectively selected all patients who underwent radiotherapy for drug-resistant non-neoplastic focal epilepsy. Data on patient and treatment characteristics as well as follow-up were collected from the electronic health records. Consent was obtained from all patients to assess and publish anonymized data prior to analysis.

### 2.2 Indication

The indication for radiotherapy was evaluated by a multidisciplinary team at our tertiary care center, including a radiation oncologist, epileptologist, neurosurgeon, and neuroradiologist. Radiotherapy was considered in and offered to patients with structural epilepsy that was both pharmacoresistant and inoperable. A recent magnetic resonance imaging (MRI) of the brain with a plausible structural defect had to be available. Informed consent was obtained from all patients prior to radiotherapy which was offered as an *ultima ratio* individualized treatment.

### 2.3 Dose-fractionation rationale

**Figure 1:**
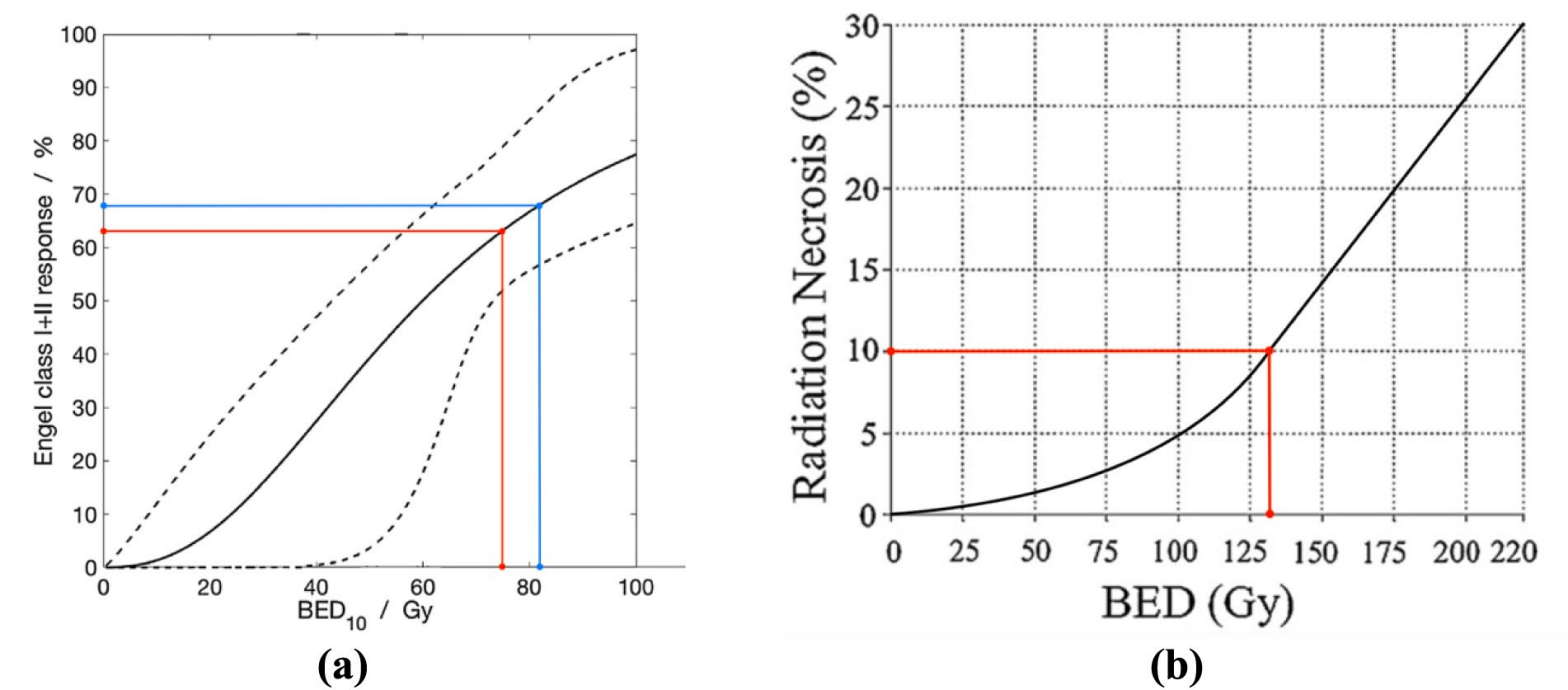
**(a)** Dose-response analysis plotting biologically effective dose with *α*/*β* = 10 (BED_10_; in Gy) versus proportion of treated patients having no or rare seizures following radiotherapy (i.e., radiotherapy-adapted Engel class I or II response; in %). The red line represents 50 Gy in 10 daily fractions of 5 Gy, the blue line represents a single SRS dose of 24 Gy. Adapted from Eekers et al. (8), with permission. **(b)** Relationship between BED (in Gy) and radiation necrosis risk (in %) after fractionated stereotactic radiotherapy. The red line represents 50 Gy in 10 daily fractions of 5 Gy. Adapted from Lawrence et al. (12), with permission.

All patients were treated with a total dose of 50 Gy in 10 daily fractions of 5 Gy, over 2 weeks, yielding a biologically effective dose of 75 Gy, considering *α*/*β* = 10 (BED_10_). In the dose-response analysis by Eekers et al. (8), this results in 60–70% of treated patients having no or rarely seizures following radiotherapy (Figure 1a). According to the Quantitative Analysis of Normal Tissue Effects in the Clinic (QUANTEC) RN risk model by Lawrence et al. (12), the RN risk associated with this dose prescription is approximately 10% (Figure 1b). In contrast, a single SRS dose of 24 Gy yields an only marginally higher BED_10_ of 81.6 Gy (Figure 1a) and thus response rate, however, more than doubles the RN risk (~20%) (8, 12). This is of particular importance when greater volumes are irradiated, which is why this risk-adapted radiotherapy concept was developed.

### 2.4 Radiotherapy planning and delivery

Patients received a simulation computer tomography (CT) in a supine position with an individual thermoplastic fixation mask for stereotactic treatments. In addition, a planning brain MRI (with at least contrast-enhanced T1-weighted and fluid-attenuated inversion recovery [FLAIR] sequences) with 1 mm slice thickness was obtained and rigidly co-registered with the planning CT. Unlike most oncological radiotherapy indications, there are no consensus-based contouring guidelines or atlases available to delineate the epileptogenic zone. Here, the target volume was defined by the multidisciplinary team. All available clinical and diagnostic (both non-invasive and invasive) information were considered. The epileptogenic zone was delineated as the gross tumour volume (GTV) with no expansion for the planning target volume (PTV). Treatment calculations were performed considering a marginal 100% isodose line on the target volume. Organs at risk (OARs) were contoured following international guidelines and prespecified dose constraints for brainstem, cochlea, eye, lacrimal gland, lens, and optic pathway were adhered to (Table 1) (13). Radiotherapy was administered with stereotactic intensity-modulated techniques, employing 6–10 MV photon energies and ensuring a target volume coverage of 95–107%, as per the International Commission on Radiation Units and Measurements (ICRU) Report 50 recommendations. This was achieved through a treatment plan with an identical field and beam arrangement for each fraction, delivering 5 Gy to the entire PTV per fraction, from different directions. Number and set-up of fields and arcs were individualized for each patient, depending on the location of the PTV and proximity to OARs (Supplementary Figure 1). All patients were treated on a TrueBeam STx linear accelerator (Varian Medical Systems, Palo Alto, CA, United States), performing daily image guidance with ExacTrac (Brainlab, Munich, Germany) for position matching.

**Table 1:** Prespecified dose constraints for relevant organs at risk (OARs) using hypofractionated stereotactic radiotherapy in 10 fractions (13).

OAR	Dose constraint
Brainstem	D_<5ccm_ < 32 Gy
Cochlea	D_<0.5ccm_ < 25 Gy
Eye	D_mean_ < 26 Gy
Lacrimal gland	D_<1ccm_ < 14.1 Gy
Lens	D_max_ < 7 Gy
Optic pathway	D_<0.5ccm_ < 30.6 Gy

### 2.5 Outcome and toxicity

Outcomes were assessed using the radiotherapy-adapted Engel classification (RAEC) (Table 2) (8, 14). Radiation-induced acute and late toxicities were assessed using the National Cancer Institute’s (NCI) Common Terminology Criteria for Adverse Events (CTCAE) version 5.0 (15). Follow-up was performed by both the treating radiation oncologist and epileptologist and included a detailed history and neurological assessment, with any additional examinations [e.g., regular contrast-enhanced brain MRI including perfusion sequences, electroencephalogram (EEG), neuropsychological testing] performed at their discretion. Neuropsychological testing was performed for the following cognitive domains: verbal skills, verbal memory, figural memory, visuospatial functions, motor skills, attention and concentration, as well as behavior (including mood), as previously described (17). Test performances in the named domains were scored on a five-category scale based on normative test results from a healthy control population corrected for age.

**Table 2:** The radiotherapy-adapted Engel classification (RAEC) (8, 14).

RAEC	Definition
I	Seizure-free
II	Rarely seizures
III	Improved >75%
IV	No significant improvement

## 3 Results

Between February 2021 and April 2024, three patients were treated with this dose-fractionation concept. Key clinical and dosimetric characteristics are summarized in Table 3. All prespecified dose constraints were met in all patients. Detailed case descriptions are reported below.

**Table 3:** Key clinical and dosimetric characteristics of included patients (*n* = 3).

Sex	Age at fSRT	Epilepsy	Location	MRI findings	EEG findings	PTV (cm^3^)	Total number of ASMs (currently taking)	Time between onset and fSRT (mo)	Follow-up (mo)	RAEC (response)	fSRT side effects
Male	47	Post-hemorrhagic lesion causing bilateral tonic-clonic seizures without aura (4–6×/yr)	Right frontolateral	Pre: Parenchymal posthemorrhagic defect with perifocal gliosis Post: Perifocal post-radiogenic gliotic changes, increased FLAIR	Pre: Intermittent regional slowing (theta and delta) frontotemporal on the right side, intermittent regional slowing (theta) over both temporal regions, more pronounced on the left side, no interictal epileptic activity, two focal to bilateral tonic-clonic seizures with onset right frontocentral (F4 and Cz) recorded Post: No epileptic activity, intermittent high-amplitude theta activity pronounced over the bilateral frontal regions	16.5	5 (2)	29	29	I	fatigue (G1), alopecia (G1)
Female	42	Rasmussen encephalitis, *epilepsia partialis continua* involving the right side of the face, bilateral tonic-clonic seizures (1×/yr), focal seizures with sensory symptoms, and/or speech arrest (2–3×/wk)	Face area of the left postcentral region	Pre: Hemiatrophy of the left hemisphere with gliosis, most pronounced in the left parietooccipital region, new onset FLAIR hyperintensity in the left postcentral gyrus Post: Constant gliotic changes, increased FLAIR	Pre: Continuous multiregional theta- and delta-slowing over the left hemisphere, variable regional maximum (frontocentral, temporoparietooccipital, etc.), interictal epileptic activity left occipital, temporooccipital, temporoparietal and temporal, seizures with mild symptoms, partially with left frontocentral onset, partially without rhythmic ictal pattern, *epilepsia partialis continua* of the right facial muscles Post: Continuous multiregional slowing over the left hemisphere with interictal epileptic activity left frontocentrotemporal, temporal, and temporooccipital, subclinical seizures with left frontal, temporooccipital and temporoparietooccipital onset recorded, intensity of *epilepsia partialis continua* significantly reduced	10.3	13 (4)	21 ^a^	36	III ^b^	fatigue (G1), alopecia (G1)
Female	45	Suspected focal cortical dysplasia causing hyperkinetic movements, crying, complex automatisms, swallowing (up to 15×/d; 2–4×/n)	Right anterior insular region	Pre: Cortical thickening and blurred white-gray matter junction Post: Constant cortical thickening, increased FLAIR	Pre: Intermittent slowing, low-amplitude spikes and sharp-waves frontocentrotemporal on the right side, (sometimes bilateral), numerous sleep-related seizures with frontal and occasionally temporal semiologic elements recorded, ictal patterns with diffuse EEG flattening, further evolution variable, rhythmic activity sometimes frontocentral right, central right, centroparietal right, frontocentral left, frontocentral on both sides, sometimes no clear ictal pattern visible, on invasive diagnostics interictal activity with rhythmic spike-waves and polyspikes on perilesional contacts, numerous seizures recorded: initially high-amplitude polyspikes, flattening, later on rhythmic low-amplitude beta-activity and low voltage fast activity (LVFA) on perilesional contacts, further evolution with polyspikes Post: Interictal epileptic activity over the frontocentrotemporal region substantially reduced, rare interictal epileptic activity in the right temporal region, recorded seizures with reduced intensity, sometimes without rhythmic ictal activity, sometimes ictal activity over the right frontotemporal region	11.3	8 (4)	264	12	III	Dizziness (grade 1), headaches (grade 1), alopecia (grade 1)

fSRT, fractionated stereotactic radiotherapy; MRI, magnetic resonance imaging; EEG, electroencephalogram; PTV, planning target volume; ASM, antiseizure medication; mo, months; RAEC, radiotherapy-adapted Engel classification; yr, year; FLAIR, fluid-attenuated inversion recovery; G, grade; wk, week; d, day; n, night.

a Onset of epilepsia partialis continua.

b Related specifically to epilepsia partialis continua.

The first patient is a 47-year-old male with a history of subarachnoid hemorrhage from a dissecting aneurysm of the V4 segment of the vertebral artery, invasively managed with a flow diverter. Upon removal of the periprocedural external ventricular drainage, an intracranial hemorrhage occurred, leaving a right frontolateral post-hemorrhagic lesion. Ever since, epileptic episodes were bilateral tonic-clonic seizures without aura, occurring around four to six times per year. Epileptic seizures had already led to multiple vertebral fractures, broken teeth, and several soft tissue wounds. Subsequently, the patient developed anxiety and was severely restricted in his activities and job, impairing his quality of life. By the time of the first presentation at the Department of Radiotherapy, five different ASMs had been tried, two of which were currently being taken. ASM-induced side effects included sleeplessness, agitation, mood swings, liver toxicity, and osteoporosis. Neuropsychological examination revealed no cognitive deficits. Verbal memory appeared to be above average, however, a depressed mood could be verified. Upon neurosurgical evaluation, the patient was deemed inoperable based on a clotting disorder.

**Figure 2:**
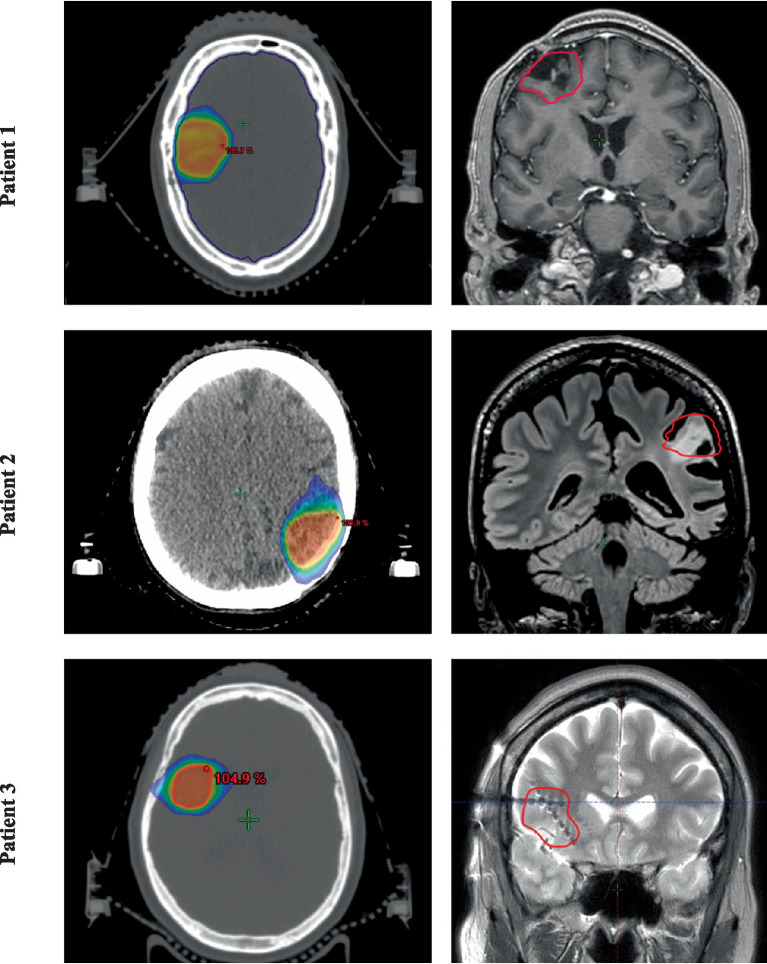
Target volume and treatment planning for all three patients. (Left) Axial fractionated stereotactic radiotherapy plan, showing the 50% isodose line for all three patients. (Right) Superimposed coronal brain MRI used to accurately define the target volume (red).

Twenty-nine months after his first epileptic seizure, fSRT was evaluated and initiated in this patient (Figure 2 first row). Concordant findings between seizure semiology, MRI, and the non-invasive presurgical work-up supported the hypothesis of a seizure generator in the right frontal lobe and aided in target volume delineation. Tolerability was excellent: as acute side effects, fatigue (grade 1) and local alopecia (grade 1) occurred, both resolving spontaneously over several months. Until the ninth day after fSRT completion, two more epileptic seizures occurred, which were, however, shorter, with fewer cloni, and with a faster recovery. From that point onward, the patient remained seizure-free during the entire follow-up period of 29 months. Subjectively, increased alertness, cognition, concentration, and multitasking were reported. Neuropsychological testing 12 months after fSRT revealed some decline in verbal memory (remaining in the average range, however) and an improvement in figural memory. Mood recovered, his former fulltime job was taken up again and after 1 year of seizure freedom, the patient was cleared for driving a car again, further promoting his independence. One of two ASMs was successfully tapered and then discontinued. During the entire follow-up period, no late radiation-induced side effects occurred. Focal neurological deficits were not observed. One year after fSRT completion, a brain MRI revealed a slightly increased FLAIR signal in the irradiated region, without evidence of RN. The EEG showed the absence of epileptic activity. The patient died 29 months after radiotherapy completion, most likely from a ruptured aneurysm of the proximal vertebral artery, very unlikely to be related to the fSRT.

The second patient is a 42-year-old female with onset of Rasmussen encephalitis at the age of 23. Due to the disease, she also developed a hemianopsia on the right side, as well as sensory deficits on the right side of her face and body. For the past 2 years, she had been suffering from *epilepsia partialis continua*, manifesting as continuous and conscious focal seizures involving the right side of the face (mainly the eyelids and corner of the mouth) up to the shoulder, impairing speech (articulation) and the ability to drink without a straw. Bilateral tonic-clonic seizures occurred about once per year. Subsequently, this patient was incapable of working. Over the course of her disease, high-dose corticosteroids had led to no improvement and 13 different ASMs had been attempted (of which four were currently being taken), resulting in the absence of tonic-clonic seizures, however, without influence on the *epilepsia partialis continua*. ASM side effects included hair loss, tremor, weight changes, and rash, further hampering quality of life. Imaging revealed an involvement of the left hemisphere, especially the face area of the postcentral region, with a newly onset FLAIR hyperintensity that later proved to be reversible (18). Results of MRI diagnostics in this patient have been reported previously. Due to the involvement of this eloquent zone, this patient was deemed unsuitable for epilepsy surgery. Neuropsychological evaluations shortly after the onset of *epilepsia partialis continua* revealed deficits in verbal and motor skills (mainly on the right side), as well as in the attention domain. Verbal and figural memory were unaffected, visuospatial functions were not tested. Mood scores appeared normal.

Twenty-one months after onset of *epilepsia partialis continua*, the patient was treated with fSRT (Figure 2 second row). Due to the presence of extensive gliotic tissue, target volume delineation was challenging in this patient. MRI showed a newly-onset lesion in the left postcentral gyrus, which was considered as the seizure onset zone upon concordance with the seizure semiology and topologic organization of the sensorimotor strip (face area). Again, tolerability was excellent, with only fatigue (grade 1) and local alopecia (grade 1) occurring, both slowly improving over time. Six weeks after fSRT completion, the *epilepsia partialis continua* had decreased drastically, both in frequency and intensity, now mainly involving the eyelids. The patient was able to drink normally again and speech had improved markedly, resulting in an improvement of this patient’s quality of life. Brain MRI 4 months after treatment completion showed an increased FLAIR signal in the irradiated area, which remained stable throughout follow-up. Three years after fSRT, the patient remains in follow-up with a stable improvement of symptoms. Neuropsychological testing 12 months after fSRT verified a significant decline in verbal memory, figural memory, and attention. After 36 months, figural memory displayed a recovery, verbal memory also improved again, however, did not reach the baseline preceding fSRT. Attention remained strongly impaired. Verbal skills did not show any significant changes. Motor skills were tested differently before and after fSRT. These declines in certain cognitive domains in this patient might also be related to the progressive disease or changes in pharmacotherapy, and are not necessarily related to fSRT. We did not observe any new focal neurological symptoms following fSRT. After fSRT, medication was unaltered.

The third patient is a 45-year-old female with a suspected focal cortical dysplasia of the right insular region. For more than 20 years, she experienced unconscious focal seizures, up to 15 times per day and three times per night, characterized by hyperkinetic movements, complex automatisms, crying, and swallowing (usually lasting no longer than 30 s). Impairments in activities of daily living caused her to give up her job. In total, eight different ASMs had been tried, of which four were currently being taken. ASMs had reduced the seizures during daytime, but did not, however, influence the nocturnal seizures. ASM intake was associated with headaches and impaired memory. This patient did not display any relevant focal neurological deficits, only a faint postural tremor of the hands. Due to the location of the lesion (proximity to the basal ganglia as well as branches of the medial cerebral artery), this patient was not a suitable candidate for epilepsy surgery.

The target volume and the extent of the seizure onset zone were determined using input from a previously conducted stereo-EEG with eight depth electrodes (Figure 2 third row). During fSRT, a reduction of the nocturnal seizure rate was already observed, as well as prolonged seizure-free intervals (before fSRT on average 1–2 days per week seizure-free, now intervals up to 4 days). Tolerability was excellent, with the occurrence of transient dizziness (grade 1), self-limiting headaches (grade 1; possibly related to fSRT), and local alopecia (grade 1). Twelve months after fSRT completion, the patient reported a gradual further improvement in seizure intensity and an increased number of seizure-free days. This patient remains in follow-up. Neuropsychological testing was not available for this patient due to a relevant language barrier. No new focal neurological deficits were observed following fSRT.

## 4 Discussion

Radiotherapy for drug-resistant non-neoplastic focal epilepsy has been under investigation for several decades and was initially mainly explored in the context of mesial temporal lobe epilepsy (MTLE) and hypothalamic hamartomas (HHs) (8). Over time, very diverse radiotherapy concepts have been tested, in a growing number of epileptic entities, however, mostly generating level 4 evidence. The only published level 2 evidence so far comes from the ROSE (Radiosurgery or Open Surgery for Epilepsy) trial, which randomized patients with pharmacoresistant unilateral MTLE to either standard anterior temporal lobectomy (ATL) or Gamma Knife-based SRS (24 Gy prescribed to the 100% isodose line, targeting the amygdala, anterior 2 cm of the hippocampus, and the parahippocampal gyrus) (16). Of 58 included patients (the trial was closed early due to slow accrual), seizure remission was achieved in 78% of ATL patients versus 52% of SRS patients within 2 years after randomization (*p* for non-inferiority = 0.82). Interestingly, with longer follow-up, the proportion of seizure-free patients in the SRS arm still increased, up to 74% after 3 years. Changes from baseline in verbal memory were similar between groups and both treatment options were deemed reasonably safe, with only few serious treatment-related adverse events reported. In a secondary report, incidence and severity of perimetrically-assessed visual field defects were also similar between arms. None of the patients reported subjective visual changes (19). Although ATL had a numerical advantage over SRS in terms of seizure freedom at the two-year mark, these data underpin the potential role of non-invasive radiotherapy for inoperable patients, those reluctant to epileptic surgery, or those with limited access to care. A similar conclusion was drawn by a systematic review and meta-analysis including 168 patients (13 studies) with MTLE (SRS dose ranging from 10–25 Gy) (20). Based on these results and an additional systematic literature review, the International Stereotactic Radiosurgery Society (ISRS) practice guideline concludes that SRS is an efficacious treatment to control seizures in MTLE, possibly even yielding superior neuropsychological outcomes and quality of life metrics in selected subjects compared to surgical intervention (21). Furthermore, in the context of small HHs treated with SRS, a better risk-benefit ratio is reported in comparison with surgery, especially if the HH is located in the dominant hemisphere. Here, no overall increase in the burden of adverse events was reported following SRS (21).

In the current case series, we further explore the role of radiotherapy for refractory structural epilepsy. Three patients with very diverse underlying causes of their epilepsy (post-hemorrhagic, Rasmussen encephalitis, focal cortical dysplasia) were included. All had been suffering from epileptic seizures, including regular bilateral tonic-clonic ones, for several years and a combined total of 26 ASMs had been tried, with associated side effects. Quality of life was majorly impaired in all patients prior to treatment. As the epileptic foci were rather large (median PTV 11.3 cm^3^, range 10.3–16.5 cm^3^), the risk of RN had to be considered. A biologically effective dose-fractionation concept was chosen, almost equivalent to a single SRS dose of 24 Gy in terms of seizure freedom (i.e., 60–70% of treated patients having no or rare seizures). The risk of RN, however, could theoretically be halved (10% versus >20%). In the 13 SRS studies included in the systematic review by Eekers et al. (8), the median sizes of the epileptic foci were considerably smaller (median PTV 7.3 cm^3^, range 1.7–10.6 cm^3^). Similarly, the ROSE trial reported irradiated volumes of 5.5–7.5 cm^3^ (16).

In all three patients treated with fSRT in this case series, tolerability was excellent, with radiation-induced side effects being mild (grade 1 only) and transient. During follow-up, post-fSRT brain MRI revealed a characteristic increased FLAIR signal in the irradiated regions without evidence of RN. After matured follow-up (median 29 months, range 12–36 months), improvement in both seizure frequency and severity was noted in all three patients, with one patient even experiencing complete seizure freedom after fSRT. All patients reported improvements in their quality of life and ASM reduction without the onset of new seizures was achievable in one. The RAEC currently does not consider patient-reported outcome measures, which are, however, of particular interest in this context, given the substantial burden of illness imposed by epilepsy (2). A further refinement of the current classification should therefore be considered, in order to properly reflect the impact of treatment. Interestingly, the effects of fSRT were rapid in some patients, with already noticeable changes in seizure frequency and/or intensity during or shortly after treatment. The meta-analysis by Feng et al. (20) (SRS for MTLE) reported an average of 14 months to seizure cessation. With ongoing follow-up of our patients, further improvements might thus be expected. The overall delayed onset of effect after radiotherapy, especially in comparison with epilepsy surgery, should be communicated accordingly. In the ROSE trial, for example, the seizure-free proportions at 3 months were 6 versus 81% following SRS and ATL, respectively, increasing to 74 versus 85% at 3 years.

As this is a highly individualized treatment, patients should be counseled accordingly and the potential of severe treatment-related side effects requires an in-depth discussion, including (permanent) alopecia, cognitive decline (with onset possibly already several months after radiotherapy; minimized by reducing radiation dose to the hippocampus), tumor induction (latency up to several decades), and RN (in rare cases requiring a salvage surgical intervention). As some of these side effects might develop over several years (RN most commonly occurs within 1–3 years after radiation, but later onset has been reported), long-term longitudinal follow-up is needed (12). Long-term neurological side effects are, however, unlikely (22, 23). fSRT cannot be recommended for children and adolescents, as they are not represented in the existing body of evidence, resulting from the well-described adverse effects of radiation on the developing brain. Appropriate patient selection and multidisciplinarity are essential when treating patients. Here, heterogeneous underlying etiologies were treated with distinct seizure phenotypes, including a post-hemorrhagic lesion, Rasmussen encephalitis, and focal cortical dysplasia. All patients had a defined anatomical substrate and reported an improvement from baseline, implying that the latter might be more important than the exact cause of the epilepsy when selecting patients. Multidisciplinarity is key when defining the target volume, as the true seizure onset zone might be different from a proposed anatomical substrate. Concordance of different examination modalities (e.g., MRI, EEG, seizure semiology) can support the location of the true target. The inclusion of a small margin of healthy brain tissue around the (MRI-visible) lesion, analogous to the neurosurgical concept of supramarginal resection, might be justified in some cases to achieve optimal outcome and prevent geographic miss. Additional clinical research is needed to identify patient or epilepsy characteristics that might predict favorable response and potentially guide clinical decision-making.

The pathophysiological effects of radiotherapy on seizure activity are still poorly understood, especially when considering that radiotherapy itself can also increase seizure activity, e.g., in the context of high-grade glioma (24). Animal models of epilepsy (usually rodents) have been evaluated to elucidate the mechanisms behind the anticonvulsant effects of radiotherapy, however, rat brains are remarkably radioresistant, preventing a direct translation of dose-fractionation concepts to the human brain (25). Notably, destruction of the epileptogenic zone is not necessary to achieve an anticonvulsant effect (26–28). In epileptic rats, a dose-dependent reduction in seizures was observed despite the lack of gross neuronal injury on autopsy. Furthermore, cognitive functions were spared (i.e., water maze performance in this context) and remained unimpaired after treatment (26, 27). These findings further strengthen our hypothesis that the homogeneous non-ablative fSRT dose-fractionation concept used in this trial yields biologically equivalent outcomes compared to SRS. Suppression of the epileptic activity through neuromodulatory effects, preferentially influencing epileptogenic rather than unaffected cortex, might restore the neurotransmitter equilibrium and lead to clinical benefits (25, 29). These effects seem to be more important than mere destruction of the epileptogenic zone and its pathways through necrotizing SRS (25).

As high-level evidence for radiotherapy of epileptogenic zones has been lacking, the prospective randomized waitlist-controlled PRECISION trial (NCT05182437) was launched (30). Eligible adults, regardless of underlying seizure-causing pathology, are randomized to a single linac-based SRS treatment (with an isotoxic dose of 24 Gy to the 100% surrounding isodose) or ASM continuation and/or neuromodulation. A maximum PTV is not defined, V_12Gy_ should, however, be <10 cm^3^. Primary outcome will be the reduction of seizure frequency per RAEC after 2 years, with study completion expected in 2028. Based on our previous experiences with fSRT presented here, a prospective single-arm evaluation (DRKS00035095) is currently being conducted, with a structured and long-term follow-up incorporating clinical, radiological, and patient-reported outcome measures.

## 5 Conclusion

Non-invasive frameless fSRT of 50 Gy in 10 fractions is feasible and potentially safe and effective in patients with drug-resistant focal epilepsy and led to improvements in quality of life. The presented novel and radiobiologically substantiated dose-fractionation concept holds promise in well-selected patients with large target volumes. A prospective single-arm evaluation with interdisciplinary structured and long-term follow-up is currently being conducted. Future randomized trials should consider comparing SRS/fSRT with other treatment modalities.

## Data Availability

The raw data supporting the conclusions of this article will be made available by the authors, without undue reservation.

## Supplementary material

SUPPLEMENTARY FIGURE 1 Treatment plan for patient 1, showing set-up fields (yellow) and four non-coplanar rotational arcs (red). The red histograms reflect the intensity of the radiation beam at the different gantry angles. This plan delivers 50 Gy in 10 identical fractions of 5 Gy to the epileptogenic zone (pink). Number and set-up of fields and arcs are individualized for each patient, depending on the location of the epileptogenic zone and proximity to organs at risk.

## References

[1] Fiest KM Sauro KM Wiebe S Patten SB Kwon C-S Dykeman J. Prevalence and incidence of epilepsy: a systematic review and meta-analysis of international studies. Neurology. (2017) 88:296–303. doi: 10.1212/WNL.0000000000003509, PMID: 27986877 PMC5272794

[2] Ioannou P Foster DL Sander JW Dupont S Gil-Nagel A Drogon O’Flaherty E. The burden of epilepsy and unmet need in people with focal seizures. Brain Behav. (2022) 12:e2589. doi: 10.1002/brb3.2589 36017757 PMC9480957

[3] Yogarajah M Mula M. Social cognition, psychiatric comorbidities, and quality of life in adults with epilepsy. Epilepsy Behav. (2019) 100:106321. doi: 10.1016/j.yebeh.2019.05.017, PMID: 31253548

[4] Keezer MR Sisodiya SM Sander JW. Comorbidities of epilepsy: current concepts and future perspectives. Lancet Neurol. (2016) 15:106–15. doi: 10.1016/S1474-4422(15)00225-2, PMID: 26549780

[5] Surges R von Wrede R Porschen T Elger CE. Knowledge of sudden unexpected death in epilepsy (SUDEP) among 372 patients attending a German tertiary epilepsy center. Epilepsy Behav. (2018) 80:360–4. doi: 10.1016/j.yebeh.2017.11.036, PMID: 29454605

[6] Thijs RD Surges R O’Brien TJ Sander JW. Epilepsy in adults. Lancet. (2019) 393:689–701. doi: 10.1016/S0140-6736(18)32596-0 30686584

[7] Jobst BC Cascino GD. Resective epilepsy surgery for drug-resistant focal epilepsy: a review. JAMA. (2015) 313:285–93. doi: 10.1001/jama.2014.17426, PMID: 25602999

[8] Eekers DBP Pijnappel EN Schijns OEMG Colon A Hoeben A Zindler JD. Evidence on the efficacy of primary radiosurgery or stereotactic radiotherapy for drug-resistant non-neoplastic focal epilepsy in adults: a systematic review. Seizure. (2018) 55:83–92. doi: 10.1016/j.seizure.2018.01.009, PMID: 29414140

[9] Vellayappan B Lim-Fat MJ Kotecha R De Salles A Fariselli L Levivier M. A systematic review informing the management of symptomatic brain radiation necrosis after stereotactic radiosurgery and international stereotactic radiosurgery society recommendations. Int J Radiat Oncol Biol Phys. (2024) 118:14–28. doi: 10.1016/j.ijrobp.2023.07.015, PMID: 37482137

[10] Dejonckheere CS Scafa D Käsmann L Zeyen T Potthoff AL Schäfer N. Boswellia serrata for the management of radiation-induced cerebral edema and necrosis: a systematic meta-narrative review of clinical evidence. Adv Radiat Oncol. (2025) 10:101732. doi: 10.1016/j.adro.2025.101732, PMID: 40092573 PMC11904484

[11] Minniti G Clarke E Lanzetta G Osti MF Trasimeni G Bozzao A. Stereotactic radiosurgery for brain metastases: analysis of outcome and risk of brain radionecrosis. Radiat Oncol. (2011) 6:48. doi: 10.1186/1748-717X-6-48, PMID: 21575163 PMC3108308

[12] Lawrence YR Li XA el Naqa I Hahn CA Marks LB Merchant TE. Radiation dose-volume effects in the brain. Int J Radiat Oncol. (2010) 76:S20–7. doi: 10.1016/j.ijrobp.2009.02.091 PMC3554255 20171513

[13] Timmerman R. A story of hypofractionation and the table on the wall. Int J Radiat Oncol Biol Phys. (2022) 112:4–21. doi: 10.1016/j.ijrobp.2021.09.027, PMID: 34919882

[14] Engel J Jr. Clinical neurophysiology, neuroimaging, and the surgical treatment of epilepsy. Curr Opin Neurol. (1993) 6:240–9. 8481567

[15] U.S. Department of Health and Human Services. (2017). Common terminology criteria for adverse events (CTCAE) version 5.0. Available online at: https://ctep.cancer.gov/protocoldevelopment/electronic_applications/docs/ctcae_v5_quick_reference_5x7.pdf

[16] Barbaro NM Quigg M Ward MM Chang EF Broshek DK Langfitt JT. Radiosurgery versus open surgery for mesial temporal lobe epilepsy: the randomized, controlled ROSE trial. Epilepsia. (2018) 59:1198–207. doi: 10.1111/epi.14045, PMID: 29600809

[17] Delev D Taube J Helmstaedter C Hakvoort K Grote A Clusmann H. Surgery for temporal lobe epilepsy in the elderly: improving quality of life despite cognitive impairment. Seizure. (2020) 79:112–119. doi: 10.1016/j.seizure.2020.05.003, PMID: 32464533

[18] Schidlowski M Bauer T David B Bitzer F Ostermann L Racz A. Ictal hypoperfusion and iron deposition in the symptomatogenic zone of epilepsia partialis continua - a case report. Seizure. (2021) 89:56–58. doi: 10.1016/j.seizure.2021.04.019, PMID: 34015570

[19] Quigg M Barbaro NM Ward MM Chang EF Broshek DK Langfitt JT. Visual field defects after radiosurgery versus temporal lobectomy for mesial temporal lobe epilepsy: findings of the ROSE trial. Seizure. (2018) 63:62–7. doi: 10.1016/j.seizure.2018.10.017, PMID: 30408713 PMC6413861

[20] Feng E-S Sui C-B Wang T-X Sun G-L. Stereotactic radiosurgery for the treatment of mesial temporal lobe epilepsy. Acta Neurol Scand. (2016) 134:442–51. doi: 10.1111/ane.12562, PMID: 26846702

[21] McGonigal A Sahgal A De Salles A Hayashi M Levivier M Ma L. Radiosurgery for epilepsy: systematic review and international stereotactic radiosurgery society (ISRS) practice guideline. Epilepsy Res. (2017) 137:123–31. doi: 10.1016/j.eplepsyres.2017.08.016, PMID: 28939289

[22] Rauch C Semrau S Fietkau R Rampp S Kasper B Stefan H. Long-term experience with fractionated stereotactic radiotherapy in pharmacoresistant epilepsy: neurological and MRI changes. Epilepsy Res. (2012) 99:14–20. doi: 10.1016/j.eplepsyres.2011.10.036, PMID: 22130038

[23] Boström JP Delev D Quesada C Widman G Vatter H Elger CE. Low-dose radiosurgery or hypofractionated stereotactic radiotherapy as treatment option in refractory epilepsy due to epileptogenic lesions in eloquent areas—preliminary report of feasibility and safety. Seizure. (2016) 36:57–62. doi: 10.1016/j.seizure.2016.02.010, PMID: 26950169

[24] Rades D Witteler J Trillenberg P Olbrich D Schild SE Tvilsted S. Increasing seizure activity during radiation treatment for high-grade gliomas - final results of a prospective interventional study. In Vivo. (2022) 36:2308–13. doi: 10.21873/invivo.12961, PMID: 36099095 PMC9463928

[25] Quigg M Rolston J Barbaro NM. Radiosurgery for epilepsy: clinical experience and potential antiepileptic mechanisms. Epilepsia. (2012) 53:7–15. doi: 10.1111/j.1528-1167.2011.03339.x, PMID: 22191545 PMC3519388

[26] Maesawa S Kondziolka D Balzer J Fellows W Dixon E Lunsford LD. The behavioral and electroencephalographic effects of stereotactic radiosurgery for the treatment of epilepsy evaluated in the rat kainic acid model. Stereotact Funct Neurosurg. (1999) 73:115. doi: 10.1159/000029766, PMID: 10853113

[27] Maesawa S Kondziolka D Dixon CE Balzer J Fellows W Lunsford LD. Subnecrotic stereotactic radiosurgery controlling epilepsy produced by kainic acid injection in rats. J Neurosurg. (2000) 93:1033–40. doi: 10.3171/jns.2000.93.6.1033, PMID: 11117846

[28] Chen ZF Kamiryo T Henson SL Yamamoto H Bertram EH Schottler F. Anticonvulsant effects of gamma surgery in a model of chronic spontaneous limbic epilepsy in rats. J Neurosurg. (2001) 94:270–80. doi: 10.3171/jns.2001.94.2.0270, PMID: 11213965

[29] Romanelli P Anschel DJ. Radiosurgery for epilepsy. Lancet Neurol. (2006) 5:613–20. doi: 10.1016/S1474-4422(06)70496-3, PMID: 16781991

[30] Zegers CML Swinnen A Roumen C Hoffmann AL Troost EGC van Asch CJJ. High-precision stereotactic irradiation for focal drug-resistant epilepsy versus standard treatment: a randomized waitlist-controlled trial (the PRECISION trial). Trials. (2024) 25:334. doi: 10.1186/s13063-024-08168-9, PMID: 38773643 PMC11106873

