# Supplementary figures and images for: Fractionated stereotactic radiotherapy in people with drug-resistant focal epilepsy: first-in-human experience with a healthy tissue-preserving dose-fractionation concept
Source: Front Neurol. 2025 Jun 26;16:1600381. doi: 10.3389/fneur.2025.1600381 (PMC12243607; doi:10.3389/fneur.2025.1600381)

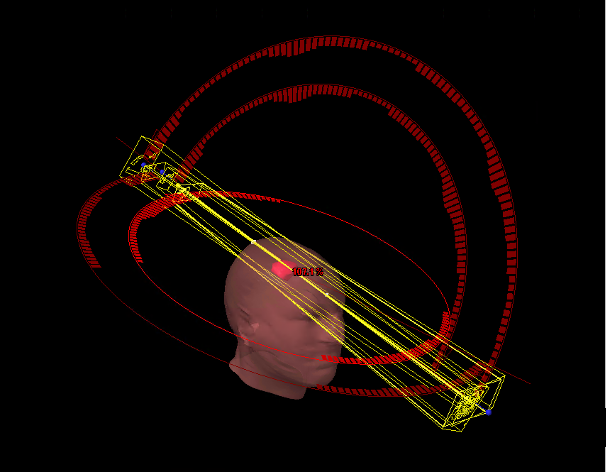

Supplement: SUPPLEMENTARY FIGURE 1 — Treatment plan for patient 1, showing set-up fields (yellow) and four non-coplanar rotational arcs (red). The red histograms reflect the intensity of the radiation beam at the different gantry angles. This plan delivers 50 Gy in 10 identical fractions of 5 Gy to the epileptogenic zone (pink). Number and set-up of fields and arcs are individualized for each patient, depending on the location of the epileptogenic zone and proximity to organs at risk. [file Image_1.png]
